# Air pollution in Mongolia

**DOI:** 10.2471/BLT.19.020219

**Published:** 2019-02-01

## Abstract

Starting in May, the government of Mongolia will introduce a coal burning ban in the capital, Ulaanbaatar, as part of efforts to clean up the city’s air. Implementing the ban is going to be a challenge, but reducing air pollution is of fundamental importance to improving population health. Sophie Cousins reports.

In June of last year, Dr Rokho Kim, an environmental specialist for the World Health Organization’s (WHO) Western Pacific Region, visited a school on the outskirts of Mongolia’s capital, Ulaanbaatar. 

“I was concerned about the proximity of schools to sources of pollution,” he says. When he got to the school, he found that it stood right next to one of the low-pressure boilers used to heat the city’s hydraulic grid. “The boilers are heated using raw coal,” he explains. “The chimney for this particular boiler was just few metres from the school’s fence. In the winter time the smoke must have been unbearable.”

A central Asian country bordered by China and Russia, Mongolia is known for its vast tracts of largely empty grassland, freezing winters and nomadic culture. In recent years it has become known for something else: some of the world’s worst air in the winter months. 

The most polluted air in Mongolia is found in Ulaanbaatar, where 46% of the country’s population resides. 

The defining characteristic of air pollution in Mongolia – as in many countries – is the high concentration of particulate matter. Measured in micrograms (millionth of a gram) per cubic metre, particulate matter consists of a complex mixture of solid and liquid particles suspended in air, and comprises a wide range of substances, from sulphates to black carbon. 

Particles with a diameter of 10 microns or less can penetrate the lungs, but the most harmful are those with a diameter of 2.5 microns or less. Such fine particles can cross the lung barrier and enter the blood system. Fine particulate pollution has health impacts even at very low concentrations. 

Ulaanbaatar’s air pollution problem has grown with the city, which has almost tripled in size since 1990, and today accommodates just under 1.5 million people. The way it has grown is as important as the extent of its growth, with intensive rural-to-urban migration resulting in a sharp increase of informal settlements. These settlements are comprised of structures called ‘gers’ – portable, circular dwellings made of wood and canvas that are insulated with felt. Ger districts, located in the north of the city, are now home to more than 60% of Ulaanbaatar’s population.

“Gers are heated with traditional stoves which stand in the centre of the structure and are connected to a chimney that passes up through the roof,” explains Dr Delgermaa Vanya, health and environment officer at the WHO Mongolia office. “These stoves can burn coal, wood, and dung, but during the winter, when temperatures can drop to −40 °C, coal is used because it burns longer than other fuels.” 

“The coal used in the stoves is a primary cause of Ulaanbaatar’s air pollution, much worse than other sources of pollution such as cars and trucks or waste burning,” Vanya says, citing a World Health Organization Regional Office for the Western Pacific policy brief published in 2018. The brief states that 80% of Ulaanbaatar’s air pollution in the winter months is caused by households and low pressure boilers burning raw coal in ger districts. 

Referred to as raw coal, the fuel burned is not washed or processed in any way and produces copious amounts of particulate matter as well as sulfur dioxide, carbon monoxide and nitrogen oxide. Dug out of the ground in the city’s Nalaikh area, it is also very cheap. 

“There is really no affordable alternative in terms of clean fuel,” says Vanya. “As a result, in the winter months over 600 000 tonnes of raw coal are burned for heating in the city’s approximately 200 000 gers, accounting for about 80% of Ulaanbaatar’s winter pollution.” 

The Mongolian National Agency for Meteorology and Environment Monitoring reports that in 2017, in the winter months that extend from November to March, the mean concentration of particulate matter for the country as a whole was between 80-140 micrograms per cubic meter. In ger districts of Ulaanbaatar, the concentration of fine particulate matter can reach well above 1 000 micrograms per cubic meter.

 Mongolia has a population of 3 million people and in 2016 an estimated 1 800 people died from diseases attributable to household air pollution and a further 1 500 people died from diseases attributable to outdoor air pollution. These included: ischaemic heart disease; stroke; lung cancer; acute low respiratory infections and chronic obstructive pulmonary disease. 

Rokho is keen to stress that the impact of pollution goes beyond the respiratory tract and lungs. “The main consequences of air pollution are not just a bad cough; they are heart attacks, and strokes. There is also emerging evidence regarding neurodevelopmental diseases, and adverse birth outcomes, and the relation between early exposure and later non-communicable diseases, such as diabetes,” he says.

Bataa Chuluunbaatar, health specialist at UNICEF Mongolia concurs, saying, “Air pollution affects all the organs that are supplied by blood vessels. It affects brain development, lung function, and the cardiovascular system.” Chuluunbaatar believes that children are particularly vulnerable to particulate matter as their bodies develop, and points to possible evidence that the finest particulate matter can pass through the placenta to an unborn child.

The 2017 *Mongolia national program on reduction of air and environmental pollution* aims by 2025 to decrease air pollutants by 80% and calls for prohibiting the use of unprocessed coal everywhere except in thermal power plants in Ulaanbaatar.

In the past, efforts to curb pollution have focused on rehousing ger district residents in apartments connected to the communal heating grid. More recently, the government started discussing the feasibility of connecting existing homes to city utilities. Subsidies have also been offered to families encouraging them to purchase stoves that produce less pollution. Since January 2017, electricity in many of the city’s highest-polluting districts has been provided free of charge at night. Needless to say, this has little impact on households not connected to electricity.

To support the government’s anti-pollution efforts, in February 2018 WHO released a set of suggested actions. 

Suggested short-term actions included a ban on the burning of waste as fuel and improving indoor air quality by banning smoking indoors. WHO also suggested improving ventilation in gers and other homes and the use of better insulation to reduce the need for heating. The creation of a sustainable support scheme to help low-income groups adopt affordable cleaner technology was also proposed.

“The coal used in the stoves is a primary cause of Ulaanbaatar’s air pollution.”

Delgermaa Vanya

A key medium-term suggestion was for Mongolia to introduce more stringent national standards for outdoor air quality, while suggestions for the long term included the drawing up of plans for the sustainable development of clean energy options. 

Just under a year later, there has been some progress. For example, the government passed a law on hygiene in 2016 prohibiting the burning of all types of waste. There has also been an initiative to encourage people to move out of the gers into better insulated and less polluting apartments. 

According Shagdar Urantsetseg, the officer-in-charge of environmental health in the Department of Public Health at Mongolia’s Ministry of Health, a fund had been set up to give those who live in gers or small houses in designated ‘air quality improvement zones’ the first 30% of their mortgage towards purchasing a new apartment. To date, some 86 households have moved to newly built apartments. More transfers of this kind will be required to have an impact on the 200 000 or so gers in the city.

The outright ban on the burning of unprocessed coal will come into effect in May in six central districts of the capital and will cover the use of raw coal in households, companies and enterprises, except enterprises with special licenses for energy and electricity generation. 

The government has also committed to providing households in ger districts with cleaner processed solid fuels and plans on having 600 000 tonnes of such fuel stored by September 2019. 

Dr Sergey Diorditsa, WHO Representative to Mongolia, welcomes the government’s commitment to ban the use of raw coal. “The challenge is going to be making sure the ban is implemented effectively,” he says. 

Diorditsa argues that, in the long term, the country will need to develop alternative energy sources. “It is important for Mongolia to continue to think how to introduce clean energy sources and technologies, and this may mean transitioning from fossil energy to renewable energy, using solar or wind power,” he says. 

Diorditsa also stresses the importance of communities and government working together on improving housing in ger areas. When the next winter rolls around, better insulated housing will make a huge difference to the heating needs of the residents of Ulaanbaatar and to the air they are obliged to breathe.

**Figure:**
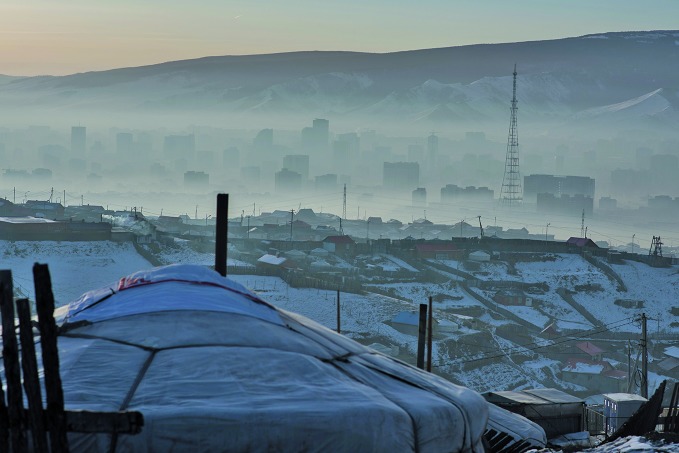
Ger district of Bayangol and Songinokhairkhan districts, Ulaanbaatar city, Mongolia.

UNICEF/Tamir Bayarsaikhan

**Figure:**
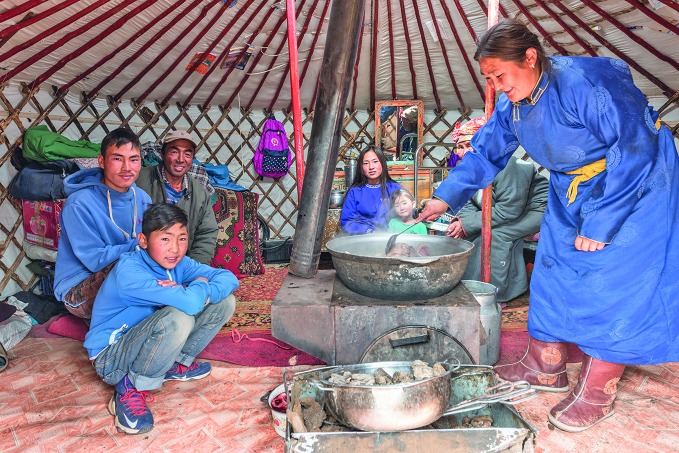
Mongolian family inside a traditional Mongolian dwelling.

UNICEF/Eric Portas Pasquall

